# Past and Present in the Ecological Connectivity of Protected Areas Through Land Cover and Graph-Based Metrics

**DOI:** 10.1007/s00267-025-02206-1

**Published:** 2025-06-17

**Authors:** Antonio Vidal-Llamas, Carolina Acuña-Alonso, Xana Álvarez

**Affiliations:** Universidade de Vigo, Hydro-Forestry Geomodeling Research Group, School of Forestry Engineering, 36005 Pontevedra, Spain

**Keywords:** Citizen science, iNaturalist, Landsat, Random forest, Biodiversity, Natura 2000

## Abstract

Habitat reduction is significantly threatening biodiversity, making ecological connectivity which facilitates species movement across habitat patches, essential for human impacts mitigation, promoting genetic exchange, and enabling colonization of new areas. Ecological connectivity in the River Lérez Basin (Galicia, NW Spain), including three Natura 2000 sites: River Lérez, Serra do Cando, and Serra do Candán, was assessed. Land cover maps for the years 2013 and 2023 were created using Landsat 8-9 images and the random forest machine learning method. Hardwood forest habitat patches and ecological corridors were identified. Betweenness Centrality (BC) metric, along with other global structural connectivity indices such as Integral Connectivity Index (IIC), Connectivity Probability (CP), and Equivalent Connectivity (EC), were calculated. iNaturalist records were then downloaded to observe how the distribution of records resembled that of habitat and corridors for 2023. Some indices, such as the splitting patch index (SPLIT), effective meshsize (EM), edge length (EL), and edge density (ED), were also calculated at the patch level. Results showed that the area of hardwoods increased by 2.16% across the basin. The spatial patch patterns were similar in both years. Additionally, hardwood structural connectivity appeared to have improved over the study period, with IIC rising in 20.00%, PC increase by 16.67–18.92%, and EC 8.04–8.68%. However, some patches from 2013 had higher BC values due to the loss of certain connections. iNaturalist record distribution showed similarities with habitat patch and path distribution, with a lower average distance (406.06 m) compared to random points (854.12 m) for 2023. The patch indices indicated a reduction in fragmentation, with SPLIT decreasing by 53.95%, EM and EL increasing by 173.66% and 14.21%, respectively, while ED decreased by 9.29%. The integration of satellite imagery has been proven effective for generating land cover data for connectivity analysis. It also demonstrates that indices and graph-based approaches offer a data-efficient alternative to traditional models. Furthermore, the incorporation of citizen science platforms, despite some biases, complements technical methods by providing real-world insights into species distribution. This combination is considered a promising approach for future research.

## Introduction

Ecological connectivity defined as the ease or difficulty of movement of the organism across a landscape (Taylor et al., 1993) is vital for the maintenance and dispersal of genes, species and populations. Moreover, diverse and well connected environments tend to own a higher stability and resilience against disturbances (Cardinale et al., 2012). In this context, the protection of biodiversity hotspots through protected areas plays a fundamental role in nature conservation, but the lack of connectivity between these areas can turn them into isolated ecosystems (Wudu et al., 2023). Despite the existence of protected areas networks, biodiversity is currently undergoing a significant decline, with 28% of assessed species by the International Union for Conservation of Nature (IUCN) under some king of threat, and 48% of animal species are experiencing population decline (Finn et al., 2023).

Preserving biodiversity is significant both from ethical considerations and practical human needs. As there is a great evidence on the close relationship between biodiversity and ecosystem services (EME, 2012; IPBES, 2019), providing essential goods, benefits and ensuring ecosystem functionality among others. In this way, biodiversity damages have a negative impact on human well-being as well. Therefore, understanding and managing ecological connectivity is indispensable, on the mitigation and resilience against environmental impacts driven by human activity and climate change (Haddad et al., 2025; Habibullah et al., 2022).

However, ecological connectivity is increasingly compromised by human-driven pressures that reshape landscapes and disrupt habitat continuity. Among these anthropogenic disturbances, land use and land cover changes (LULC), which is one of the main global change drivers (Verburg et al., 2011) and is related with other phenomenon as urban development, rural abandonment, agricultural changes or wildfires between others, implies changes in ecological connectivity and habitat fragmentation (Salazar et al., 2021; Gülçin & Yılmaz, 2020). Therefore, it has a consequent impact on biodiversity, ecosystem functioning and human well-being.

In the study area, as in many other regions with a high degree of land fragmentation, the combination of scattered landscapes (Ónega López et al., 2015), and processes such as rural depopulation (López-Iglesias et al., 2013) has created significant challenges for territorial planning and biodiversity conservation. In addition, although Spain is a pioneer country in terms of protected areas to preserve his natural values, historically, Galicia has always been at the bottom regarding the establishment and conservation of protected areas (Cabalar et al., 2016), in fact is the autonomous community with a lowest protected surface, which is in contrast to the fact that is one of the largest forested areas in Spain, with around a 60% of surface covered by forest, which represents 11% of the national total (Marey-Pérez et al., 2014). Given this context, the preservation of the limited protected areas and the reinforcement of ecological connectivity among them are considered essential to prevent further habitat degradation.

Given the acknowledged importance of ecological connectivity in land management and conservation, several methodologies have been developed. Among them, methods based on populations and metapopulations modeling approach often require a large amount of data, are usually necessary information on abundance, demographic structure among others (Calabrese & Fagan, 2004; Daniel et al., 2023). Although, it has the potential to provide finer results than other methods, being the most complete and detailed, commonly there is not the previous data require and need for a great field effort, this means that it is not always possible to use these approaches. In contrast, structural connectivity index is not needed for species-level data, and it is possible to get an idea only with a habitat map. If we also have information about dispersion capacity and patch occupancy, this allows us to work with graph theory, with spatially explicit and more detailed results than the indexes (Calabrese & Fagan, 2004), this information is not usually difficult to find through literature and gives an insight into potential connectivity. In this way, graph based methods are able to deliver similar results to population-based models with higher cost-effectiveness and a good balance between data requirements, complexity, and ecological relevance (Daniel et al., 2023). This potential is evidenced by its increasing use in the literature, and its application in a wide range of environments and species (Foltête et al., 2020).

Citizen science presence records provide an opportunity to complement graph-based studies and the identification of habitat patches. Just a really small amount of studies use citizen science platforms data as a presence data source for ecological connectivity assessment (e.g. Rueda-Uribe et al., 2024; Serret et al., 2022). Citizen science initiatives such as iNaturalist, despite their biases, not only can help increase the knowledge of species distribution useful in connectivity analysis, but also raise awareness of conservation and the relevance of scientific research (Saunders et al., 2018). Reasons why more citizen science projectors are emerging and being institutionally promoted (Vohland et al., 2021). This innovative approach opens new avenues for enhancing ecological connectivity assessments with real-world species presence data, bridging the gap between distribution models and ground truth.

For this reason, the purpose of this study is to assess the ecological connectivity evolution in the River Lérez Basin, which includes three protected areas: the River Lérez riparian forest, Serra do Cando and Serra do Candán. The territory is also characterized by a high degree of land fragmentation and several environmental pressures, including the presence of non-native tree species and recurrent wildfires. To address this, satellite imagery and remote sensing technologies—an expanding field of research—are employed. In addition, citizen science platforms represent a promising yet underutilized source of biological data. These platforms not only encourage public participation but also provide innovative information whose potential remains largely unexplored (Della Rocca et al., 2024) offering a new approach for ecological connectivity studies and a valuable solution to data scarcity. The expected outcomes include an updated analysis of land cover dynamics and connectivity trends across the basin, as well as the spatial identification of functional ecological corridors, habitat patches, and conservation priority areas. These results will deliver actionable knowledge to support spatial planning and environmental governance in the province of Pontevedra, contributing to the strategic management of its protected area network. Moreover, the applied methodology holds potential for replication in other ecologically sensitive regions facing similar challenges (Daniel et al., 2023).

## Literature Review: Landscape Fragmentation And Graph-based Connectivity Metrics

Habitat fragmentation refers to the process by which large and continuous habitats are divided into smaller patches, usually due to human activities (Valente et al., 2023; Fahrig, 2003). However, there is no consensus on the relationship between landscape fragmentation and its biodiversity effects, as they can be variable and there are differences in the preferences of each species. Habitat specialist or highly habitat-dependent species may be threatened by fragmentation, while more generalist species may benefit (Fahrig et al., 2019; Chetcuti et al., 2020).

Nevertheless, fragmentation can lead to the isolation of populations and associated negative effects. To assess fragmentation, several indices have been developed, including the splitting patch index (SPLIT), which reflects the degree of division of habitat patches; effective meshsize (EM), which indicates the average area of the connected patches; edge length (EL), which refers to the total length of the patch boundaries; and edge density (ED), which expresses the edge length relative to the total habitat area (Soifer et al., 2021; Jaeger, 2000). These indices are frequently used to assess fragmentation, for instance, Chetcuti et al. (2020) used SPLIT and EL to assess how fragmentation affects species richness on the Tibetan Plateau, Lawrence et al. (2021) used EM to assess the fragmentation of the whole Nature 200 network, Su et al. (2022) used landscape attributes such as ED to observe their effect on fungal diversity.

Ecological connectivity can be assessed by several approaches. Structural connectivity is based solely on the size, shape, and distribution of habitat patches, while functional connectivity takes into account species behavior and their actual movement capacities (Holyoak, 2019). In this context, graph-based methods serve as a bridge between both concepts, being spatially explicit and incorporating basic assumptions about species movement without requiring exhaustive data (Holyoak, 2019). The usefulness of this approach is demonstrated by its use in a variety of locations and scenarios. For example Sahraoui et al. (2021) asses the ecological networks of different target species in southwestern France thought equivalent connectivity (EC) and interaction flux, while Campos et al. (2020) studied the connectivity of amphibians in the Brazilian Atlantic forest through graphs and probability of connectivity (PC).

In addition, the application of structural connectivity or fragmentation metrics such as Integral Index of Connectivity (IIC) (Pascual-Hortal & Saura, 2006), PC (Saura & Pascual-Hortal, 2007) and EC (Saura et al., 2010) provide valuable general information on the overall connectivity of the study areas. As is evidence in studies that use this general metrics to asses ecological connectivity, Rao et al. (2025) applied PC to assess the structural ecological connectivity in a metropolitan area, Zhang et al. (2019) evaluated the connectivity of urban green areas though IIC and several scenarios, while Correa Ayram et al. (2017) tackled the anthropogenic impact on habitat connectivity in a Mexican diverse landscape through EC.

These metrics can be further refined by graph-based techniques that allow for the identification of ecological corridors and key habitat nodes using indicators such as betweenness centrality (BC). Although these approaches are less detailed than full functional models, they require fewer data and offer a cost-effective option for landscape-scale assessments. It should be noted, however, that they rely on simplifying assumptions, such as uniform dispersal distances, which may vary even among populations of the same species or at different times (Santini et al., 2013). Additionally, landscape conditions are not static and change over time, which implies the need for monitoring over the years. In summary, the reviewed approaches provide a robust theoretical and methodological basis for assessing landscape fragmentation and ecological connectivity. The application of fragmentation indices and connectivity metrics in the present study allows for a spatially explicit, data-efficient analysis of connectivity patterns in the River Lérez Basin. This framework supports informed conservation planning in the context of limited data availability, land-use change, and biodiversity pressures.

## Methods

### Study Area

**Fig. 1:**
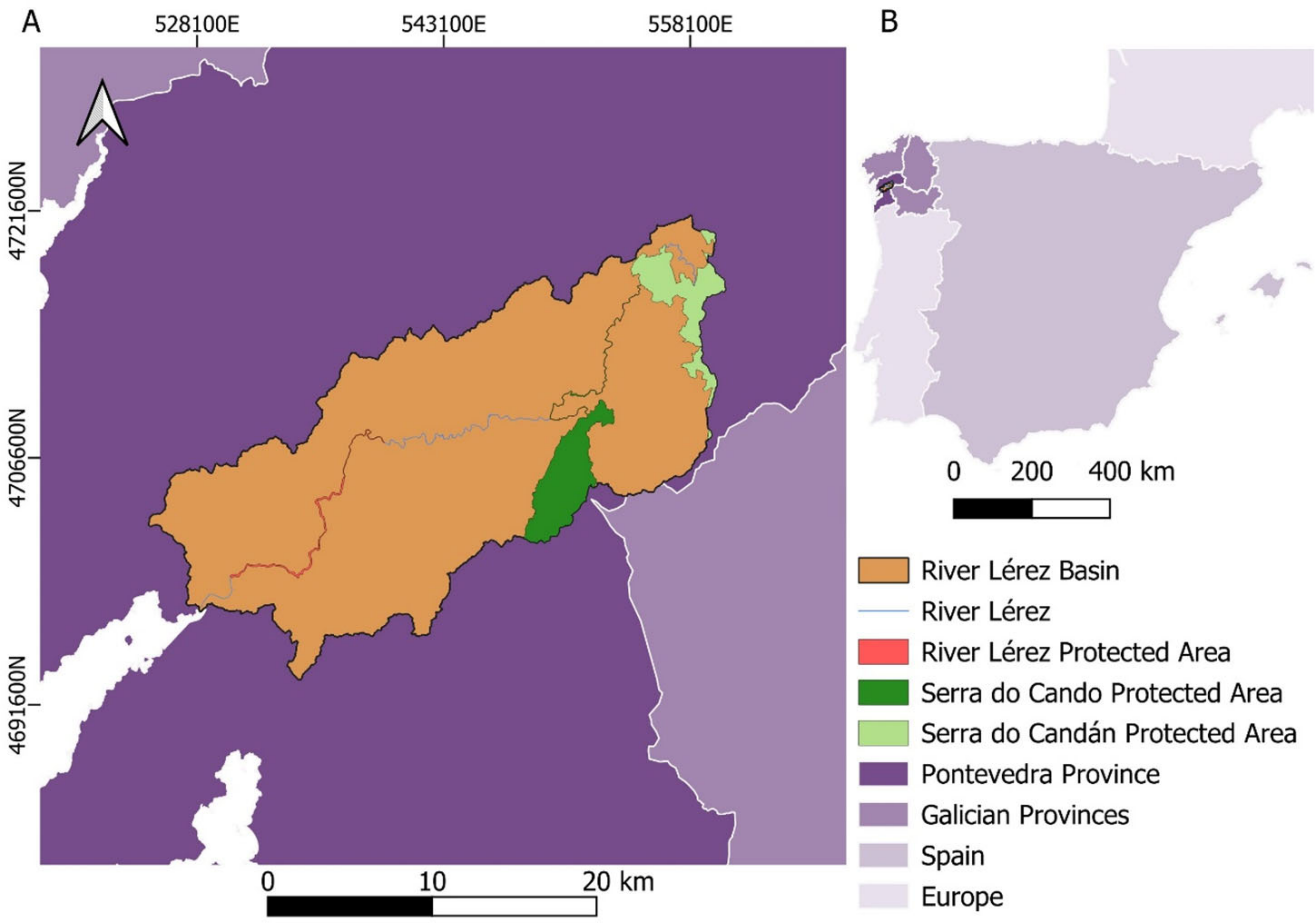
**a** Location of the River Lérez Basin within the province of Pontevedra. **b** Location of the province of Pontevedra and the rest of the Galician provinces. The map coordinate system is EPSG:25829 ETRS:89/UTM zone 30

The study area selected was the River Lérez Basin, which is located in Galicia, northwestern Spain (Fig. 1). With a surface of 450.99 km^2^ a significant portion (8.19%) is under European protection through habitats directive (BOE, 1992), three protected areas are located, River Lérez (ES1140002) in its full extension (1.50 km^2^), Serra do Cando (ES1140014, 19.44 km^2^) and Serra do Candán (ES1140013, 16.04 km^2^), both partially included in the area of the basin under study. There are several population centers distributed throughout the basin, however population tends to concentrate on the southwest, close to the sea and the mouth where Pontevedra city is located with 82.592 inhabitants (INE, 2024). The averages annual temperature swing between 12.10 °C (Forcarei) and 15.66 °C (Pontevedra). However, it varies seasonally, average temperatures during the cold season (December to February) varies between 7.67 °C (Forcarei) and 10.85 °C (Pontevedra), while it ranges between 16.87 °C (Forcarei) and 20.89 °C (Pontevedra) in the warm season (June to August). Moreover, average annual precipitation ranges between 1656 mm (Pontevedra) to 2503 mm (Forcarei), with the highest rainfall in the cold season where average monthly precipitation is between 214 mm (Pontevedra) and 333 mm (Forcarei) compared to 39 mm (Pontevedra) and 68 mm (Forcarei) along the warm season (MeteoGalicia, 2024). The altitude varies from sea level on the mouth to 1000.11 m.a.s.l on Serra do Cando, Serra do Candan is another relevant high spot with altitudes usually exceeding 800 m.a.s.l (IGN, 2024) this represents an east-west altitude gradient on the basin. Most of the basin is under colline bioclimatic zone with exception of the highest points, which belong to montane zones coincided with the areas of Serra do Cando and Serra do Candan (Meteosierra, 2023). Regarding lithology is dominated by fascies, with exception of mountainous areas which are composed by schist (IGME, 2024).

According to Land Occupation Information System of Spain (SIOSE, by its initials in Spanish) (IGN, 2014), the basin is dominated by agricultural and herbaceous land (48.28%) and hardwood vegetation (27.57%), while mix forest (12.63%), conifers (6.62%), artificial (4.58%) and water bodies (0.30%) does not cover such large surfaces. The territory is marked by a high degree of fragmentation (Franco, 2011), which is a challenge for land connectivity. The three protected areas have a variety of community interest habitats (Xunta de Galicia, 2012). Throughout the basin, there is a great Surface of oak forest dominated by *Quercus robur*. Although with a smaller surface, riparian forests are dominated by *Alnus glutinosa*. Besides tree-covered areas, there is also great surfaces of shrubby vegetation where *Ulex europaenus* and *Erica spp* are the most common covering great part of Serra do Cando and Serra do Candán (Gobierno de España, 2024). Regarding non-natural vegetation there is notorious the presence of *Eucalyptus globulus* crops which is related with several environmental issues (Deus et al., 2022) and some surfaces of *Pinus pinaster* crops. Not only eucalyptus that is of concern, is known the presence of invasive alien trees as *Acacia dealbata* (Vázquez de la Cueva et al., 2022) and *Acacia melanoxylon* (Jiménez et al., 2008). Then there is others environmental issues as the frequency and intensity of wildfires (Driscoll et al., 2021) and the intention to build a new highway which is in conflict with the River Lérez protected area conservation. The presence of non-native vegetation as eucalyptus or invasive alien species as *Acacia dealbata* and *Acacia melanoxylon* (BOE, 2013) are combined wildfires and the probability of a new highway are some of the main threats to ecological connectivity and environmental governance in the basin.

### Conceptual Framework

**Fig. 2:**
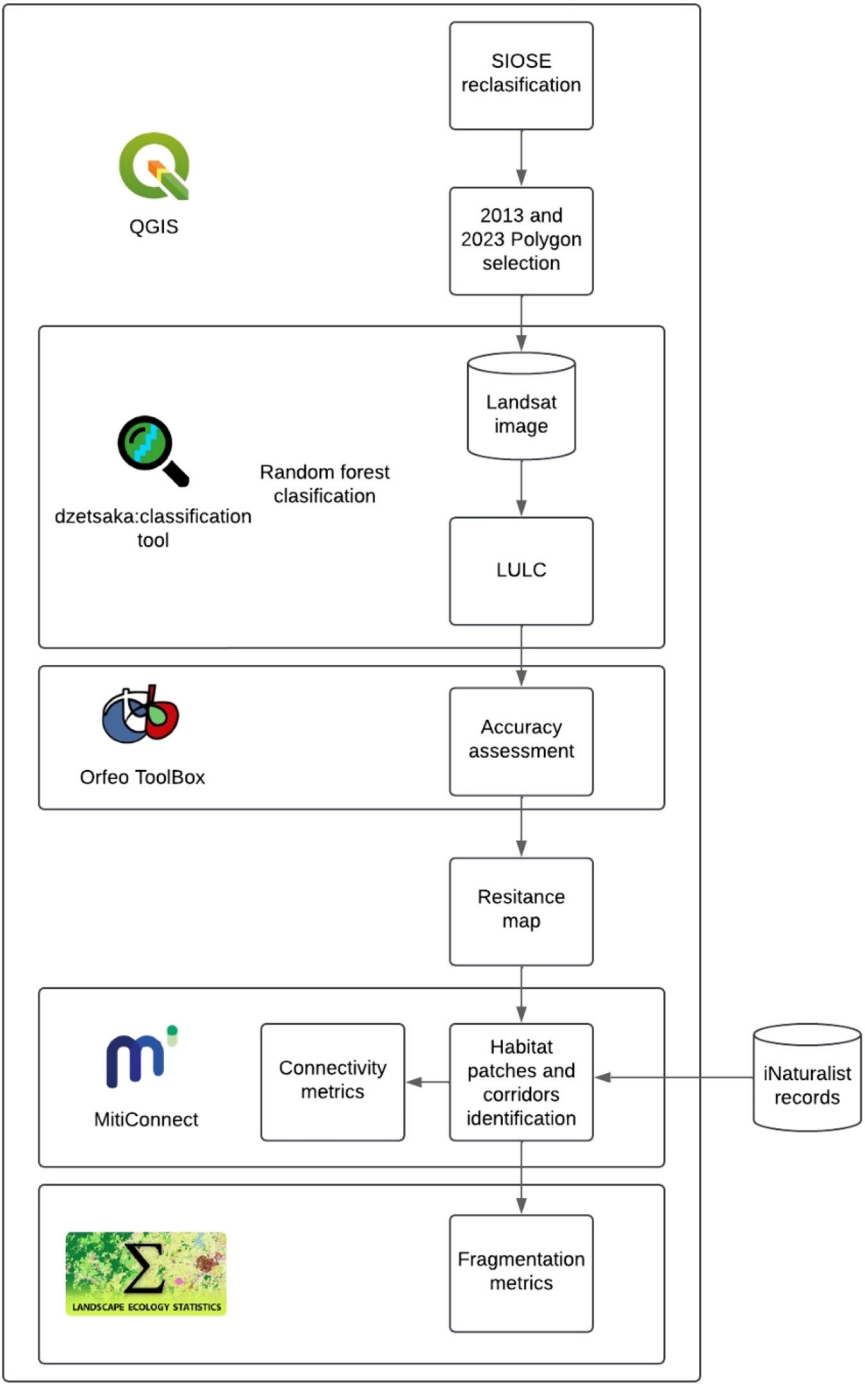
Workflow of the method used in the study. LULC Land Use and Land Cover, SIOSE Land Occupation Information System of Spain

The method used (Fig. 2) comprises two land covers classifications through Landsat 8-9 images and random forest (RF) performed with the dzetsaka plugin using as a reference a SIOSE reclassification, resistance maps created through the land cover classification and road and train layers. With the MitiConnect plugin, land cover results and resistance maps were used to identify potential habitat patches, corridors and calculate connectivity metrics. In addition, a record species distribution was downloaded from iNaturalist in order to analyses the coincidence between users’ observation and patches and corridors pattern. The whole study was performed on the open-source software QGIS (QGIS, 2024).

### Image Collection and Land Cover Classification

Two Landsat 8-9 images were downloaded provided by the United States Geological Survey (USGS) through EarthExplorer website (USGS, 2024) corresponding to Augst 2013 and July 2023, both with a 30-m resolution, an atmospheric correction already established and less than 10% of clouds. The classes to be considered for classification were agricultural and herbaceous, artificial, conifers, hardwoods, eucalyptus and water bodies. The classes were reclassified based on SIOSE (Table 1, Appendix S1).

**Table 1:** Structure of a confusion matrix with associated metrics for classification accuracy evaluation

			Predicted class		
Validated class		Class 1		Class 2	Total
	Class 1	*C*_*11*_		*C*_*12*_	*R*_*1*_
	Class 2	*C*_*21*_		*C*_*22*_	*R*_*2*_
	Total	*P*_*1*_		*P*_*2*_	*N*
	Commission error	$1-\frac{{C}_{11}}{{P}_{1}}$		$1-\frac{{C}_{22}}{{P}_{2}}$	
	Omission error	$1-\frac{{C}_{11}}{{R}_{1}}$		$1-\frac{{C}_{22}}{{R}_{2}}$	
	Accuracy				$\frac{\mathop{\sum }\limits_{{\rm{i}}=1}^{{\rm{n}}}{C}_{{\rm{i}},{\rm{i}}}}{N}$
	Kappa index				$\frac{N\bullet \mathop{\sum }\limits_{{\rm{i}}=1}^{{\rm{n}}}{C}_{{\rm{i}},{\rm{i}}}-\mathop{\sum }\limits_{{\rm{i}}=1}^{{\rm{n}}}{R}_{{\rm{i}}.}\bullet {C}_{.{\rm{i}}}}{{{\rm{N}}}^{2}-\mathop{\sum }\limits_{{\rm{i}}=1}^{{\rm{n}}}{R}_{{\rm{i}}.}\bullet {P}_{.{\rm{i}}}}$

Where, *N* is the total of validation pixels, *C*_*i,i*_ are the pixels on the diagonal (correct predictions), *R*_*i*_ is the sum of the pixels in a row _*i*_, *C*_*i*_ is the sum of the pixels in a column _*i*_ and *n* is the number of classes.

Classification was made selecting training polygons for each class in QGIS and was performed through random forest method. RF is a machine learning algorithm consisting of a collection of tree-structured classifiers, where each classifier is created using random vectors and each tree cast a unit vote for the most voted class (Breiman, 2001), which has proven to obtain good levels of accuracy regarding other methods (Pal, 2005). Polygons were delineated through photointerpretation, supplemented by verification using Google Street View to ensure accurate classification. Classification was executed with the dzetsaka plugin in QGIS (Karasiak, 2016).

### Accuracy Assessment of LULC Classifications

To ensure the quality of the classification, validation polygons were selected through photointerpretation and Google street, covering 0.31% of the area in 2013 and 0.26% in 2023 which is significant according to literature (Colditz, 2015) although there is no although there is not fixed correct surface. Subsequently, a confusion matrix was generated, and the Kappa index was calculated (Carletta, 1996) (Table 1) to assess classification accuracy thought the QGIS plugin Orfeo toolbox (OTB).

### Ecological Connectivity

For the connectivity analysis it is necessary to take into account reference species, small mammals are considered appropriate and frequently used in this type of study. We know that several species of mustelids are present and the species *Martes foina* and *Meles meles* are the most frequent in the study area, according to GBIF (GBIF : The Global Biodiversity Information Facility, 2024). In accordance with Virgós & García (2002), who studied the populations of *Martes foina* in Spain, a maximum dispersion of 5 km and a patch size of 20 ha has been considered, although it could vary according to individual species and habitat quality.

In order to assess the ecological connectivity must be taken into account the landscape permeability. So through our land cover layers, a road layer and a train layer (Xunta de Galicia, 2024) we build a friction layer based on Gobierno Vasco (2016), which made a methodological proposal (Table 2), a buffer of 30 m to roads and railways and 40 m for highways were applied.

**Table 2:** Resistance to passage through land cover classes, railways, roads and highways

Coverage	Resistance
Agricultural and herbaceous	20
Artificial	1000
Conifers	10
Hardwoods	1
Eucalyptus	20
Water bodies	100
Railway	150
Roads	100
Highways	1000

Through the recently released plugin MitiConnect (Chailloux et al., 2024) in QGIS available from March 2024, habitat patches were identified, was calculated the least-cost paths network and a graph map with all patches represented as nodes and edges as connections. The indicator used to evaluate the connectivity was betweenness centrality (BC) in order to identify the importance of patches (Men & Pan, 2024; Bodin & Saura, 2010). BC represents the sum of the shortest paths through a patch (i) and associate a weight depending on their connection capacity and their likelihood of interaction with other patches, it is the extent to which a patch contributes to the movements between other patches (Eq.1):

1 $${{BC}}_{i}=\sum _{J}\sum _{k}{a}_{j}^{\beta }{a}_{k}^{\beta }{e}^{-\alpha {d}_{{jk}}}$$

Where $B{C}_{i}$ is the betweenness centrality metric of a patch *i*, *j* and *k* is a pair of patches, *a* the patch area, β parameter adjusts the influence of patch size on connectivity and ${d}_{{jk}}$ is the distance between *d* and *k*.

### Global Metrics

Global metrics, including the Integral Index of Connectivity (IIC) (Men & Pan, 2024; Pascual-Hortal & Saura, 2006), Probability of Connectivity (PC) (Van Moorter et al., 2023; Saura & Pascual-Hortal, 2007) and Equivalent Connectivity (EC) (Sahraoui et al., 2021; Saura et al., 2010), were also calculated to assess connectivity across the entire basin rather than for individual patches, using the MitiConnect plugin (Chailloux et al., (2024)) in QGIS. For this purpose, dispersal distances of 1000 m, 3000 m, 5000 m, 7000 m, and 9000 m were considered. The IIC represents the overall connectivity of the map on a scale from 0 to 1 (Eq.2):

2 $${IIC}=\frac{1}{{A}^{2}}\mathop{\sum }\limits_{i=1}^{n}\mathop{\sum }\limits_{j=1}^{n}\frac{{a}_{i}{a}_{j}}{1+n{l}_{{ij}}}$$

Where ${a}_{i}$ is the area of each patch, $n{l}_{{ij}}$ the number of links and A the area of the whole landscape.

The PC expresses from 0 to 1 the probability that two random points are connected (Eq.3):

3 $${PC}=\frac{1}{{A}^{2}}\mathop{\sum }\limits_{i=1}^{n}\mathop{\sum }\limits_{j=1}^{n}{{a}_{i}a}_{j}{e}^{-\alpha {d}_{{ij}}}$$

Where ${a}_{i}$ and ${a}_{j}$ are the areas of habitat patches *i* and *j*, *d* is the distance between *i* and *j*, also *A* is the global area.

The EC refers to the area of a patch with the same connection probability as the pattern of patches in the landscape, never can be smaller than the area of the largest patch (Eq.4):

4 $${EC}=\sqrt{\mathop{\sum }\limits_{i=1}^{n}\mathop{\sum }\limits_{j=1}^{n}{{a}_{i}a}_{j}{e}^{-\alpha {d}_{{ij}}}}$$

Where ${a}_{i}$ and ${a}_{j}$ are the areas of habitat patches *i* and *j* and *d* the distance between *i* and *j*.

### Species Distribution

In order to know how species distribution is related with identified pathways and habitat patches we downloaded an iNaturalist record on 10/23/2024. iNaturalist (iNaturalist, 2024) is a citizen science social network where users upload species records, as a social network. Users could be recreative naturalist with a higher mistake rate compared with other sources, so a quality filter was applied. Specifically, records were limited to “research grade” that means that the records had location, data, evidence (picture, sound record…) and users community agree on the species identification and have a high precision (Ackland et al., 2024). As species could share paths, search was limited to mammifers overall, then non representative and livestock species were deleted, among them: *Eptesicus serotinus, Equus caballus, Erinaceus europaeus, Oryctolagus cuniculus, Ovis aries, Sciurus vulgaris*, *Talpa occidentalis* and *Tursiops truncates*. iNaturalist records, highlight the prominence of certain species along the basin, 107 records were obtained between 2008 and 2024. Among them, *Sus scrofa* (26) was the most common species, followed by *Canis Lupus* (25), *Vulpes vulpes* (23) and *Capreolus capreolus* (21), other species like *Genetta genetta* (6), *Martes foina* (3), *Meles meles* (1), *Lutra lutra* (1) and *Felis silvestris* (1) was barely observed. Regarding the time distribution of record, although most of them belonged to 2019 onwards, the first record data from 2008 (1), there was a great amount of records in 2009 (13) and 2010 (21), there was almost no more records until 2018 (3) and increase in 2019 (10) and 2020 (16), there is other decrease in 2021 (5) and 2022 (2), and increase again in 2023 (17) and 2024 (15) (Fig. 1, Appendix S1).

The records were represented as a points layer and the minimum distance to the 2023 layer of habitat patches and paths of each point was measured. A layer of 200 random points was created, and the distance of each point was measured as well in order to know the average distance of a random point. A heatmap with an influence of 5 km by record was created too in order to identify records hotspots.

### Patch Metrics

Finally, we use the identified potential habitat patches, where some metrics were calculated at the patch level to better understand the evolution of fragmentation and connectivity in the study area. These metrics were splitting patch index (SPLIT), effective mesh size (EM), edge length (EL) and edge density (ED) and were calculated through the LecoS plugin in QGIS. SPLIT is a fragmentation index that indicates how many equal portions of habitat would have to be split to obtain the same level of fragmentation (Jaeger, 2000) (Eq.5):

5 $${SPLIT}=\frac{{A}_{t}^{2}}{\mathop{\sum }\limits_{i=1}^{n}{A}_{i}^{2}}$$

Where ${A}_{t}$ is the total area of potential habitat, ${A}_{i}$ area of a patch *i* and $n$ the number of patches.

EM indicates the average area of the connected patches (Jaeger, 2000) (Eq. 6):

6 $${EM}=\frac{1}{{A}_{t}}\mathop{\sum }\limits_{i=1}^{n}{A}_{i}^{2}$$

Where ${A}_{t}$ is the total area of potential habitat patches, ${A}_{i}$ area of a patch *i* and $n$ the number of patches

EL means the total length of the edges of the patches (Eq.7):

7 $${EL}=\mathop{\sum }\limits_{i=1}^{n}{E}_{i}$$

Where ${E}_{i}$ is edge length of a patch *i* and $n$ the number of patches.

Finally, ED refers to the edge length of all patches relative to the total area of potential habitat (Soifer et al., 2021) (Eq.8):

8 $${ED}=\frac{{EL}}{{A}_{t}}{10}^{4}$$

Where ${EL}$ is the total edge length and ${A}_{t}$ is the total area of potential habitat patches.

## Results and Discussion

### Land Cover Changes Analysis

**Fig. 3:**
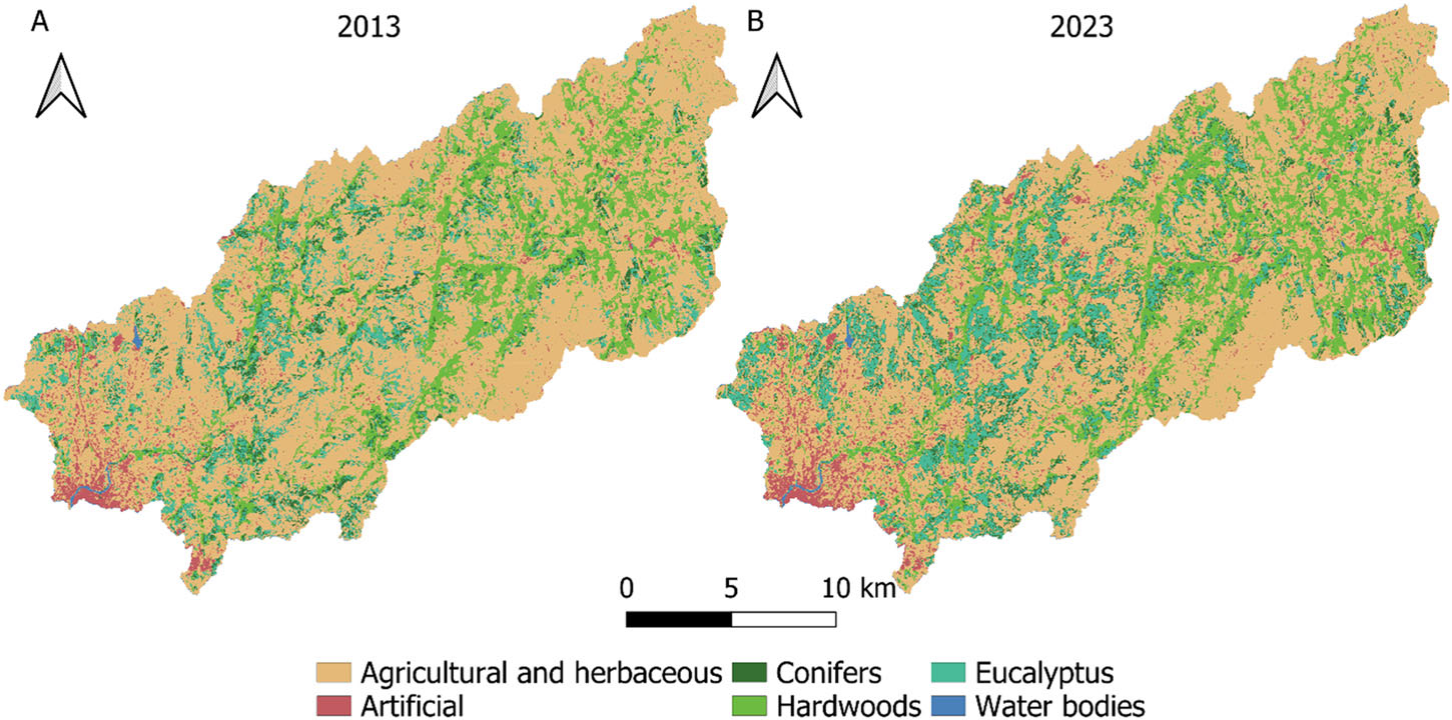
Comparison of spatial distribution of land cover classification results in 2013 **a** and 2023 **b** on the River Lérez Basin

Land cover classification results (Fig. 3, Table 3) reveal some changes on the distribution and surfaces. Regarding agricultural and herbaceous vegetation, which is the dominant class on the basin, is distributed along the whole basin, including Serra do Cando and Serra do Candán, which are mostly covered by shrubby vegetation. Furthermore, it has experienced a decrease of 7.41% in relation to the whole basin. On the other hand, with regard to the forest area, it has experienced a general increase in 6.13% considering the three classes. These results match with several studies that have observed that, it has experienced an increase in the European continent and the Iberian Peninsula over the last decades (Velázquez et al., 2022; MA, 2005). Moreover, changes in both classes could be related with rural abandonment, a particularly worrisome phenomenon in Galicia, by which the rural population abandons the countryside and moves to urban areas (Pazo & Moragón, 2017). In addition, it is necessary to highlight the increase in the area of hardwood forest (2.16%). The increase could not only be related to rural abandonment, but also the administration recognize the value of native hardwoods and it enhance them promoting its plantation (Xunta de Galicia, 2021; BOE, 2012). Furthermore, hardwood forest is mainly located around the riverbend and the east, close to the mountains of the protected areas Serra do Cando and Serra de Candán, influence of protection measures, soil and hydrological riverbends conditions would be affecting the expansion of this vegetation (King & Keim, 2019).

**Table 3:** Cover surface (km^2^) relative frequency (%) and relative change (%) of land cover classification

	2013		2023		Relative change (%)
Cover class	km^2^	%	km^2^	%	
Agricultural and herbaceous	290.74	64.47	257.34	57.06	−7.41
Artificial	15.35	3.40	21.14	4.69	+1.29
Conifers	23.83	5.28	37.77	8.37	+3.09
Hardwoods	72.66	16.11	82.38	18.27	+2.16
Eucalyptus	46.92	10.40	50.85	11.28	+0.88
Water bodies	1.48	0.33	1.51	0.33	0

**Fig. 4:**
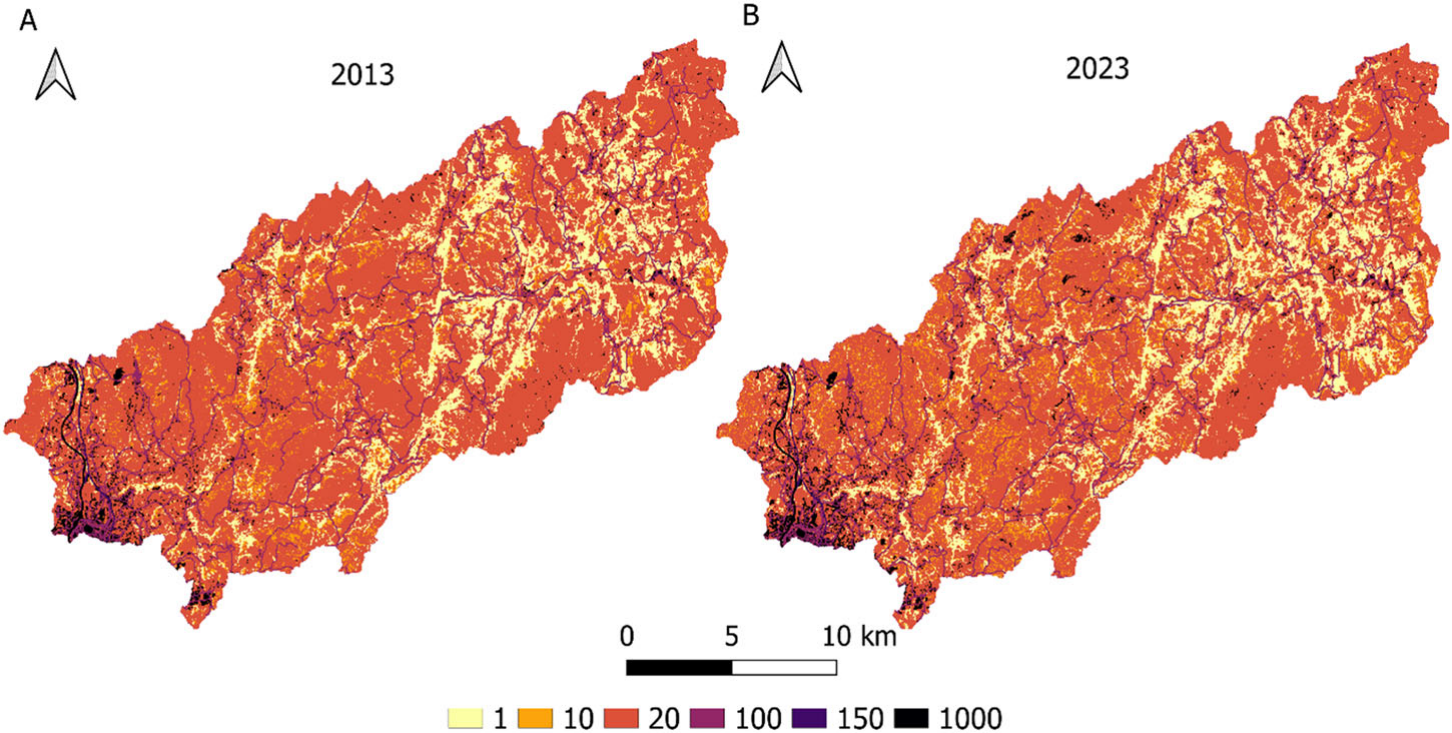
Comparative spatial distribution of ease of movement between 2013 **a** and 2023 **b**

Moreover, eucalyptus, which tends to be near to the sea and population centers has a notable surface as well and was increased by 0.88%. The eucalyptus class was added to the reclassified classes (Table 1, Appendix S1), even though it does not appear as a class in SIOSE reclassification, due to we are aware about the large area occupied by eucalyptus trees (mainly *Eucalyptus globulus*), in addition to being a non-native species with a highly invasive character (Deus et al., 2022). Eucalyptus is a controversial crop highly extended on the Atlantic biogeographic region of Iberian Peninsula (Deus et al., 2022). Despite being valuable on the local forestry industry is also associated with environmental troubles such as water consumption (Lara et al., 2021), ecosystem services reduction (Castro-Díez et al., 2021), fire hazard (Fernandes et al., 2011) or biodiversity impacts (Castro-Díez et al., 2021) between others. Even habitat fragmentation as an indirect impact as a consequence of the expansion (Calviño-Cancela et al., 2012) which can lead to connectivity reduction. This situation is particularly worrying in Galicia, as the trend on the Iberian Peninsula, according to the different climate scenarios, is for the niche to move northwards (Deus et al., 2022), making this autonomous community an even more propitious place for its expansion. Then the proximity of eucalyptus crops to protected areas is concerning, since it implies a colonization risk (Deus et al., 2022). These situation has led the local government to become aware of it with the ban on new eucalyptus plantations to stop their expansion (BOE, 2021) until end of 2025, although it could be extended. For the concerns of this study, several authors agree that the presence of small mammals and specifically mustelids is reduced in eucalyptus forests (Pereira et al., 2012; Cruz et al., 2015), lowering landscape permeability (Fig. 4, Table 2) compared with others forest surfaces. This means that growing eucalyptus in detriment of conifers or hardwoods surface would be weakening ecological connectivity.

In relation to conifer forest is not very widespread in the basin, although has been significantly increased covering a 3.09% more respect the whole basin by 2023. Regarding artificial class, despite not fill a large are area, it has increased significantly (1.29%), artificial surfaces are one of those implies graters environmental impacts and is undergoing a general increase (Lin et al., 2019). This surface is located mainly in the in the lower places, matching with the river mouth and close to the sea, where Pontevedra city is located, with 82.592 permanent inhabitants which increase during the summer is the main population center. Regarding water surfaces, although in no case it cover a significant area of the basin, as the riparian forest closes in, detection becomes impossible due to tree cover above the stream and a pixel size larger than the river width (Bolick et al., 2021), so its underestimation is inevitable.

In addition, it should be noted that the study area has the presence of Acacia dealbata and Acacia melanoxylon, both cataloged as invasive species (BOE, 2013). These species, which are scattered in small areas throughout the basin have a high flammability index (Toy-Opazo et al., 2024). The presence of these species, in an area which is the most fire-prone region in Europe (Daminatis et al., 2021) implies serious human and environmental risks. Together, these factors can lead to habitat reduction and fragmentation, increasing the risk of isolation (Driscoll et al., 2021). Moreover, in the current global change context, empty niches are often colonized by fast-growing non-native species, such as eucalyptus and acacias. These species, adapted to fire-prone environments, regenerate quickly and can outcompete native pioneer vegetation (Driscoll et al., 2021), feedback on the problem. Combined with alterations in the soil chemistry (Castro-Díez et al., 2021) and seed bank (Hernán & Bejarano, 2012) make these exotic trees a threat to native forest and local fauna.

### Land Cover Validation

With respect to validation results obtained for the year 2013 (Table 2, Appendix S1), it shows a good performance in the classification, 93.92% of the compared pixels matched. Furthermore Kappa index obtained a good score as well (0.88), that indicates a highly higher agreement than expected by a random classification and is in line with other studies (Foody, 2020). Nevertheless, not all classes behaved in the same way, agricultural and herbaceous vegetation, artificial and hardwoods were highly accurate, with low commission and omission errors. Among them, artificial class was also correctly classified with few commission and omission errors (0.02 and 0.05), although some confusion with agricultural and herbaceous surfaces was observed. Rural areas tend to have a high mosaicism between artificial and agricultural surfaces in semi-urban areas, this implies the presence of many pixels between the boundaries of both classes and surfaces whose spectral signature has characteristics of both surfaces, which could explain this confusion. In contrast, conifers and eucalyptus have a lower precision, actually 30% of pixels belonged to other classes were not correctly classified as conifers and 235 of eucalyptus pixels were classified as other classes. This confusion between eucalyptus could be affecting connectivity results considering the lower value of eucalyptus regarding other classes as conifers and hardwoods.

A solid performance in the 2023 validation results was obtained as well (Table 3, Appendix S1), 92.64% of the compared pixels matched. The Kappa index, at 0.87, also indicates a strong agreement beyond random. As in the previous classification, the agricultural and herbaceous classes obtained good accuracy and the artificial class shows confusion with the agricultural and herbaceous class, again there are discontinuous rural areas where artificial cover is in interspersed with agricultural lands where a greater confusion between these classes would be expected. Hardwoods class showed a moderate commission error (0.11) but a low omission error (0.05). In contrast to the previous classification, eucalyptus performed in a better way, maybe the training sample selected for 2023 was more representative of its spectral signature. Finally, as in 2013 conifers shows a significant commission error (0.35) with confusion with other forest classes, there is also a relevant omission mistake (0.25). Particularly some of the eucalyptus pixels belonged to conifers affecting the obtained results.

Both classifications show some errors among the three classes of tree surfaces, which is logical considering that forest surfaces are more prone to confusion among themselves than among other classes, as their spectral signatures are closer to one another (Mallinis et al., 2023).

### Ecological Connectivity and Fragmentation

The layer of resistance to movement created, shows how east resistance areas have increased by 2023 (Fig. 4), especially in the east of the basin, which suggests a permeability increase, so movement paths would be improving as a consequence of hardwood forest expansion.

**Fig. 5:**
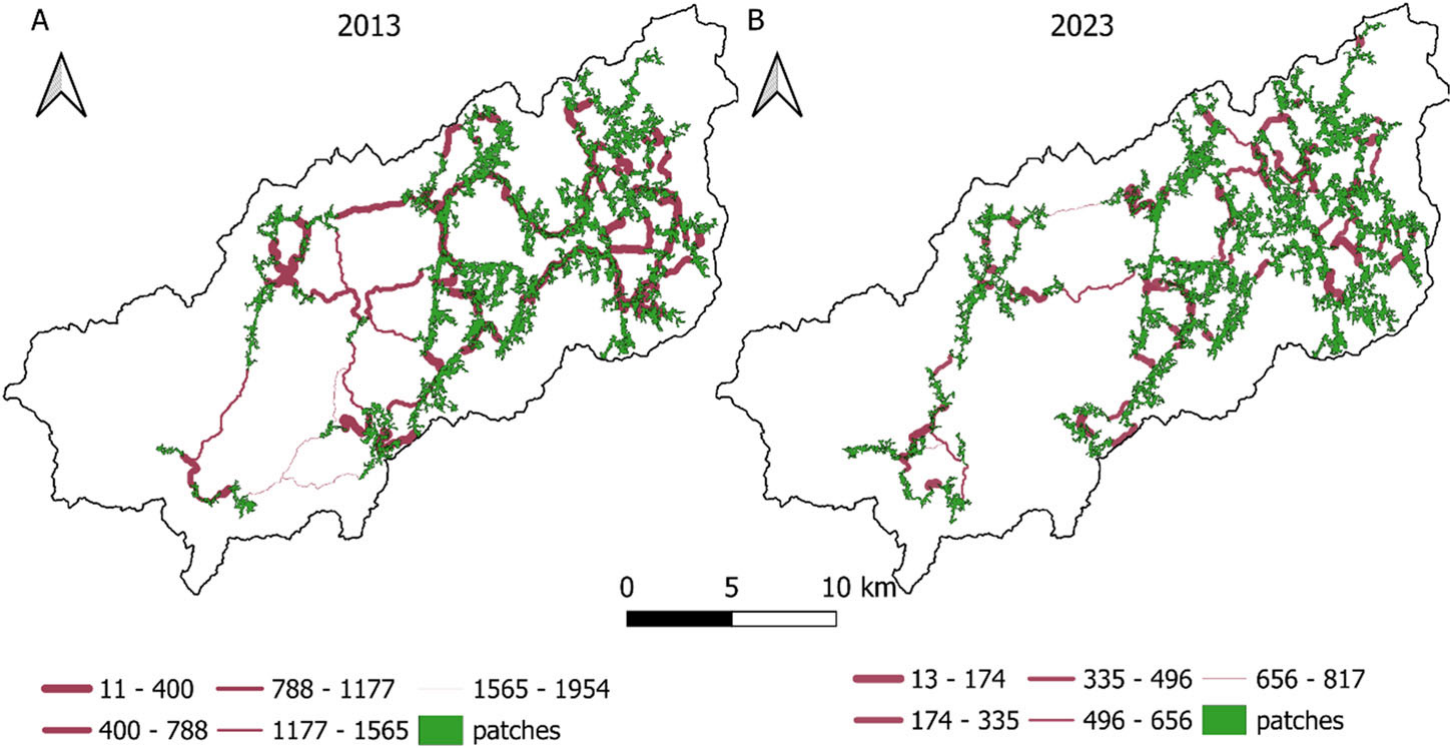
Comparative habitat patches and path pattern by effective distance and equal intervals between 2013 **a** and 2023 **b**

In 2013, 44 habitat patches have been identified (Fig. 5a), ranging between 0.21 km^2^ and 6.35 km^2^ with an average size of 1.01 km^2^ a median size of 0.59 km^2^ (Fig. 2, Appendix S1) and 44.71 km^2^ as total potential habitat. Most of the potential habitat patches, are around the center and west, where effective distance of the paths tends to be smaller, in addition there are some patches on the western zone, close to the river mouth an artificial area, this patches and patches leading from them follow a similar pattern with the riparian forest of River Lérez protected area (Fig. 1). On the other hand, by 2023 46 potential habitat patches were obtained (Fig. 5b), with sizes between 0.20 km^2^ and 4.75 km^2^, an average of 1.14, a median of 0.56 km^2^ and a total area 51.27 km^2^ (Fig. 2, Appendix S1). Results do not show some pathways that were present in 2013, although the maximum effective distance was lower. Again, most of the patches are in the central and eastern region where are the shortest paths with some more in the west which follow the river course. Furthermore, the corridors identified represent the cost distance, although this is also related to longitudinal distance and the shortest paths tend to be the strongest links.

There was found a similar pattern for both years, although some paths have been lost in 2023. These were some of the largest and weakest ones, in fact the maximum effective distance on the path network has been greatly reduced in 2023. Path pattern have strong coincidences with the main riverbend, including the riparian forest ZEC of the River Lérez protected area as the riparian forest also included in Serra do Cando that connects with Serra do Candán. Given the strong literature supporting the importance of rivers as ecological corridors (Rinaldo et al., 2018), it is not an unexpected outcome. Despite this, rivers and riparian areas are some of the ecosystems which are under a higher degradation and reduction of ecosystem services (EME, 2012), therefore its conservation must be a priority. With the exception of riparian zones, there are not too many paths in the protected areas of Serra do Cando and Serra do Candán as these are covered mainly by shrubs, and it is not common to find hardwoods forest there. These emphasize the importance of riparian forests in connectivity even when we talk about terrestrial connectivity, the riparian forests conservation favors connectivity between protected areas that have a common fluvial network.

Despite the importance of rivers and riparian forests as ecological corridors and their relevance for biodiversity, in the city of Pontevedra, part of the Gafos River, an affluent of River Lérez, was buried for the urban development of the city (García-Alén et al., 2022). The Gafos River is recognized as a local interest natural area (Xunta de Galicia, 2019), for which there are citizens’ initiatives calling for the recovery of its urban section. It is an urban stretch of about 525 m in length. While nature-based solutions, such as urban river restoration encourage urban biodiversity, recover ecosystem services from rivers, make urban environments friendlier for local wildlife and improve urban green infrastructure. If these types of measures were implemented in the study area, it would be possible to improve the ecological connectivity between such unique and nearby protected areas.

In addition, although this study is focused on terrestrial connectivity, some species with mainly aquatic behaviors such as *Lutra lutra*, which are linked to riparian forest could colonize some of them as well. Even non-native fauna as *Neovison vison* which occupies a similar niche and it is present in Galicia where there is fur farms that pose a risk to its expansion (Martínez-Rondán et al., 2017) could colonize this patches as well.

Galicia is characterized by a high landscape fragmentation (Franco, 2011). However, the relationship between fragmentation and ecological connectivity is complex, a fragmented habitat does not always mean a low potential habitat surface (Fahrig, 2003). While some species prefer highly cohesive habitats, others are able to live in border zones and even enjoy landscape mosaicism. For border species with low surface patch requirements, fragmentation is not a drawback in case the patches are not isolated, and the area of potential habitat is not reduced (Marchesan & Kolasa, 2024). The analysis of this study considers surface and distance criteria to identify habitat patches, the shape and edge length of the patches was not included. Therefore, it could be carried out in future studies to obtain conclusions in this regard.

**Fig. 6:**
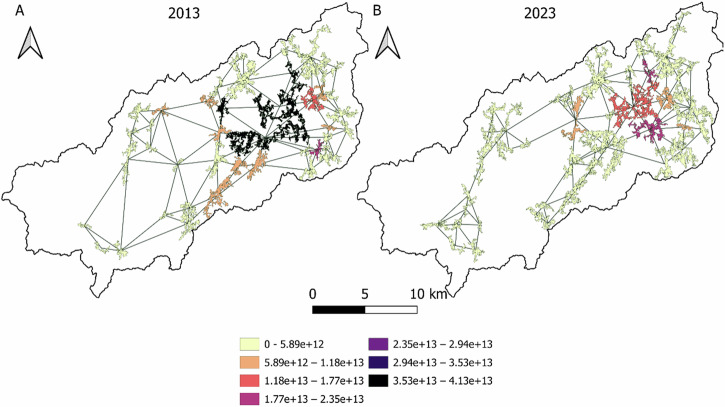
Comparative betweennesses centrality weights of habitat patches and links with other patches between 2013 **a** and 2023 **b**

Furthermore, the importance of each patch in terms of betweennesses centrality metric was observed (Fig. 6). Central nodes in 2013 obtained grater values than 2023 because of the loss of some paths. Generally, the nodes in the central regions tend to a greater BC. In contrast, western areas, with more isolated patches show a lower BC. Western patches are not only located far away from the center, which is a disadvantageous position for connection with other patches, but also artificial surfaces tend to be mainly on the west side of the basin, hindering animal movement between these patches and the rest of the basin. Largest patches tend to have a higher BC as well although this relationship is not clear (Fig. 3, Appendix S1), and the position is a more relevant issue for the BC.

This analysis is at basin level and the out-of-basin patches were not considered; however the basin is immersed in a broader landscape matrix, and some patches could be relevant for the connections with others outside of the basin. Another factor to consider is that BC weights are assigned according to the number of paths passing through a patch and their position with respect to others. Not only the position, size or connections concerns the patch relevance. The model trats the entire hardwoods forest as equal, while habitat quality should be relevant as well. There is a possibility that a well-preserved habitat may be more visited and inhabited than a more degraded one, despite its greater isolation, as the quality is related to the probability of occupancy (Santos et al., 2008) being as relevant as patch size and position (Dennis & Eales, 1997). Even a forest area that does not get the requirements to be identified as habitat patch could be chosen and colonized if there is a high quality over others that suit with the size requirements. Could be the case with some sections of the River Lérez protected area and other riparian forests areas well-preserved, which do not occupy a large area and even due to their narrow width they are not easily detectable by remote sensing. A shape criteria was also not taken into account, although patches with the same surface and different shapes would have differences in the occupancy probability according to the species preferences (Keller & Sullivan, 2023). Other authors agree that e relevance of the habitat patches and occupancy probability is in fact due to the combined effect of position, surface, habitat quality and shape (Guttery et al., 2017).

In overall, forest area has increased, resulting in an increase in the potential habitat surface, especially hardwoods. As a result, metrics show an apparent general increase in connectivity and decrease of hardwood fragmentation over the last decade, mainly related to the increase in hardwood area, although it has not resulted in an increase in BC because of the loss of some paths.

Global structural metrics show a general improvement by 2023 regarding 2013 (Table 4). IIC has increased by 20.00%, which indicates an overall connectivity gain. PC and EC both increase with greater dispersal distances and show improvements ranging from 16.67% to 18.98% and from 8.04% to 8.84% respectively.

**Table 4:** Global metrics by dispersion distance

	2013			2023					
Distance (m)	IIC	PC	EC (km^2^)	IIC	PC	EC (km^2^)	ΔIIC (%)	ΔPC (%)	ΔEC (%)
1000	0.00070	0.00048	21.74	0.00084	0.00056	23.50	+20.00	+16.67	+8.10
3000	0.00070	0.00080	28.11	0.00084	0.00094	30.53	+20.00	+17.50	+8.61
5000	0.00070	0.00099	31.21	0.00084	0.00116	33.92	+20.00	+17.17	+8.68
7000	0.00070	0.00111	33.16	0.00084	0.00132	36.09	+20.00	+18.92	+8.84
9000	0.00070	0.00121	34.52	0.00084	0.00143	37.64	+20.00	+18.18	+8.04

The three metrics applied shows an improvement on the structural hardwoods forest connectivity, Saura et al. (2010) studied the European forests and their connectivity and observed a general slight increase in connectivity between 1990 and 2000 arguing that rural abandonment could be one of the causes behind this phenomenon, rural abandonment is becoming more pronounced over the time and is a concern in Galicia (Corbelle-Rico & López-Iglesias, 2024), so this could support our results as well.

Regarding iNaturalist records do not distribute evenly along the basin (Fig. 4, Appendix S1), the main hotspot is close to Serra do Cando, on the west slope. There is also other relevant area located between Serra do Cando and Serra do Candán, which match with the main habitat patch concentration, remaining records have a more dispersal location. The further record from a habitat patch or path was 5501.21 m, the average distance between a record and a habitat patch or path was 406.06 m, the median distance was 208.01 m (Fig. 5, Appendix S1), while the average distance of a random point was 854.12 m.

A shorter average distance to landscape elements, compared to a random distribution, suggests a spatial association between species occurrences and the structure of habitats and pathways. Although a concentration of records is observed in a specific area (Fig. 4, Appendix S1) this may represent both a sampling hotspot and a true presence hotspot. It is well documented that citizen science data often exhibit spatial biases toward accessible locations such as trails (Clemente et al., 2019) and areas near population centers (Van Berkel et al., 2018), which remains a methodological challenge. Nevertheless, in our case, the distribution of iNaturalist records appears more dispersed and is not predominantly clustered around the city of Pontevedra—the only major urban center in the basin. This spatial pattern suggests broader geographic coverage and potentially reduced urban bias. Considering the growing institutional support for citizen science as a tool for biodiversity monitoring, particularly within European frameworks such as the Biodiversa+ Citizen Science Toolkit (European Biodiversity Partnership, 2021) and Horizon Europe initiatives (European Commission, 2021), evaluating the applicability and limitations of these data sources is not only relevant but necessary.

There is also notorious that most of the records belonged to big mammals, and others mesocarnivores (*Vulpes vulpes, Genetta genetta* and *Felis silvestris*) with which the mustelids have great niche similarity (Barros et al., 2024) and not mustelids. Which not necessarily mean that they are not present, there is an imperfect detection factor that affects all populations studies. It is possible corroborate the presence of an organism, not at all the absence, there is always the possibility that it has not been detected, besides the fact that no all the species are equally detectable (Mackenzie & Royle, 2005). Bigger mammals leave deeper footprints and more pronounced marks on the ground, so they are easier to detect, consequently, is highly complicated to distinguish mustelids species by the footprints or excrement. In addition, species like *Canis lupus* have a higher social value and catch more attention. Furthermore, it should be noted that the use of geolocated records implies as well certain located error which could vary according to meteorological conditions or users devices (Koo et al., 2022; Zielstra & Hochmair, 2013).

In line with the results obtained, not only is there an apparent spatial relationship between iNaturalist records and forest habitat patches, but the patch-level metrics also indicate a decrease in fragmentation and an increase in ecological connectivity over the period analyzed (Table 5). The reduction in the SPLIT index (−53.95%) and ED (−9.29%) suggest that habitat patches have become either larger or more aggregated, reflecting a less fragmented landscape. This is further supported by the increase in EM ( + 173.66%), which indicates that patches are more connected and better integrated within the landscape matrix. Although, EL, a metric often associated with fragmentation, increased in 14.21%, this likely reflects an overall expansion of forest habitat rather than increased fragmentation, as evidenced by the decrease in ED. Collectively, these trends suggest reduced fragmentation, lower patch isolation, and a consequent improvement in landscape connectivity—particularly beneficial for hardwood specialist species that are more sensitive to habitat discontinuity.

**Table 5:** Comparative patch metrics

	2013	2023	Relative change (%)
SPLIT	17.70	8.15	−53.95
EM (km^2^)	265.09	725.44	+173.66
EL (km)	1142.06	1304.34	+14.21
ED (km/km^2^)	24.33	22.07	−9.29

### General Discussion

In order to expand the temporal distance without having to mix images from several satellites and according to studies, it was decided to use Landsat due to its advantage of provide a wider temporal frame (López et al., 2024). Even in the first instance we took into account the use of Landsat 7 images in order to get a wider temporal frame. However, it was not possible to get recent Landsat 7 images without Scan Line Corrector failure (SLC) (Markham et al., 2004), a bug where empty data lines appear in the images. It should be noted that the pixel size of Landsat 8-9 is 30 ×30 m. There are other satellites such as Sentinel-2 with a higher spatial resolution of 10 × 10 m, which could be more convenient considering the fragmentation of the territory. In combination with its better spectral, it resolution could improve the classification. Nevertheless, Sentinel is a more recent satellite and only provides data since 2015 (ESA, 2015), which is the reason why it was not used. But it will certainly be a great source of information for future temporal analysis. On the other hand, the period from 2013 to 2023 could be an insufficient time period to observe significant changes in the land cover considering the surface area. Observed differences could also be explained by the classification’s confusion. Therefore, it would be interesting for future studies to extent the time period, since long-term analysis is convenient for cover changes observation (Zhong et al., 2021), although this implies the inconvenience of mixing different satellite images.

The results obtained for both classifications show good accuracy, however polygons were selected by photo interpretation with orthoimages and Google Street View, since the Landsat images were from 2013 and 2023, while the field study was in 2024, so field validation was not possible. Although fieldwork is always the ideal, the use of orthoimages combined with Google Street View is an alternative tool to reduce fieldwork when it is not possible, saving time and cost (Yeo et al., 2022). In addition, for the polygons selection we avoided using training or validation areas that could cause confusion and those where the class could not be guaranteed by photo interpretation. Although random forest is a widely used classification method because of its good results, often performing better than other classifications such as support vector machine or artificial neural networks (Belgiu & Drăgu, 2016). Despite the classification showing good accuracy, the supervision of the polygons by photo-interpretation and not by fieldwork is a limiting factor, polygons obtained in the field could increase the validation reliability.

Moreover, in order to carry out the connectivity assessment, a criterion of 5 km dispersion distance and at least 20 ha for patch habitats was used according to mustelids as a target group, which are present in the study area and are commonly used in connectivity studies for being representatives (e.g. Balestrieri et al., 2019; Fonda et al., 2021). Therefore, the results could be extrapolable to other species to the extent of their behavioral and preference similarities such as *Genetta genetta*, or other mesocarnivores which often occupy similar niches (Barros et al., 2024).

On the other hand, GBIF was considered as an option as well for the records data, however, many records were not accurate enough in the coordinates and the majority of records belonged to a single fox census. Likewise, most of the research grade iNaturalist records are usually in GBIF as well, so downloading a GBIF record and deleting inappropriate data would have been a very similar result. Given their limitations, alternative data sources, such as social networks, provide a promising data source (Minin et al., 2015). Use of social media data does not involve field effort, reducing time and cost of studies. Furthermore, they enable citizens to participate as citizen scientists (Martínez Pastur et al., 2016) encouraging public participation in decision-making processes, and raising awareness of the relevance of biodiversity and scientific research (Özden & Velibeyoğlu, 2023) among other issues, creating a bridge between citizens and researchers. Institutions have become aware of this and recognize their role and promote it, especially in issues related to environmental monitoring. The European Union recognizes the importance of public participation and awareness, especially in the invasive alien species issue (Official Journal of the European Union, 2014) and is promoting citizen science through initiatives such as the European Alien Species Information Network (European Commission, 2025), or the European Citizen Science Platform (EU-Citizen.Science, 2025). Because of that, social media data have increased their popularity around environmental issues such as ecosystem services (Oteros-Rozas et al., 2018), management (Barry, 2014), education (Potsikas et al., 2023) or alien species detection (Potsikas et al., 2023) among others. Despite some biases, given the potential of social media data, they should not be dismissed, but should be explored further (Rosário et al., 2025).

Despite the results showing an improvement regarding the increase in structural connectivity and decrease in fragmentation. The study area is subject to different environmental pressures. It will probably be affected by a highway construction (Ministerio de Fomento, 2018), linear infrastructures are a challenge for terrestrial connectivity as they have the greatest impacts on the fragmentation and isolation of populations (Ament et al., 2023), it would negatively affect the ecological connectivity in the basin. Many citizens are against its construction arguing the lack of necessity, a possible impact to the River Lérez protected area and inconveniences for the neighbors (ADEGA, 2024). Considering the project magnitude, and despite being a relevant criterion, others such as the cost-benefit of its construction, opportunity cost, impact on key species, inconvenience to neighbors or travel time reduction, should also be taken into account, in order to decide the route or the convenience of its construction. Although it could help to identify risk areas from wildlife passage. Not only linear infrastructures such as roads, urban development overall negatively impacts ecological connectivity, not only through habitat fragmentation or habitat loss, but also through the reduction of habitat quality in places close to intense human activities (Tarabon et al., 2020). Therefore, land cover monitoring and the application of possible land cover scenarios is relevant in order to anticipate possible impacts on connectivity (Wei et al., 2023). The highway construction, in combination with other pressures such as, urban development, wildfires hazard (Daminatis et al., 2021) and non-native vegetation expansion (Calviño-Cancela et al., 2012) threaten present and future connectivity. Although overall connectivity appears to have increased in terms of identification of habitat patches, there are other key factors as habitat quality and habitat patch shape, which are also relevant for occupancy probability (Guttery et al., 2017) and could be affecting actual connectivity. In fact, the patch level indices show a reduction in fragmentation, which despite having a complex relationship with connectivity is likely to be favoring it.

It is also important to note that this study is exploratory in nature, as it assesses the structural and potential connectivity based on a 6 classes land cover map, graph-based methods and connectivity metrics, which are useful tools. As an aspect of improvement, it could be complemented with other studies that focus on the analysis of different groups and habitat requirements, in order to provide a broader view. They could also be integrated with other population studies that require a large amount of data and field effort (Calabrese & Fagan, 2004; Daniel et al., 2023), if more precise results are required. However, there are some limitations, a higher time frame allows better observation in land cover changes, the use of higher resolution satellites would allow for finer analysis, and even within patches they are treated equally regardless of their conservation status. Despite its potential, the use of iNaturalist does involve some biases.

### Practical Recommendations

As an innovative aspect, this study introduces a new approach to ecological connectivity conservation, integrating structural connectivity models with citizen science tools and active participation platforms. The incorporation of social network data and citizen participation not only reduces field effort, but also fosters collective engagement in environmental decision-making, raising public awareness of the importance of biodiversity and scientific research that should be promoted. In that sense, biodiversity participatory monitoring programs in collaboration between experts and citizens such as BioBlitzes (Meeus et al., 2023) can play a relevant role in nature conservation and research. Moreover, this methodology allows for interdisciplinary and multi-stakeholder collaboration that addresses current environmental challenges from a more inclusive and sustainable perspective, positioning itself as a pioneering strategy to address the challenges of habitat fragmentation and global change (Baratella et al., 2023). According to the results, it is recommended to prioritize the conservation of the most relevant habitat patches, which usually are in the transition zone between the protected areas Serra do Cando and Serra do Candán, as well as riparian forest (Rinaldo et al., 2018), add to ecological restoration measures to enhance those paths that show a greater effective distance. Furthermore, nature-based solutions such as dentification of areas for restoration according to an ecological connectivity criterion or wildlife crossings for those areas where human development inevitably conflicts with ecological connectivity are recommended. This should be accompanied by continuous monitoring of land cover through low-cost methods such as satellite imagery and of the different pressures affecting the study area such as fires or eucalyptus plantations, particularly in areas close to protected areas or relevant patches, enabling managers and stakeholders to make informed and timely decisions, alongside the integration of ecological corridors into territorial planning.

## Conclusions

In this study, we presented an analysis of land covers changes, potential habitat patches, and ecological corridors in the River Lérez Basin (Galicia, NW Spain) between 2013 and 2023. The analysis finds some land cover changes, a general increase in forest classes conifers (3.09%), hardwoods (2.16%), eucalyptus (0.88%) and artificial surfaces (1.29%), while agricultural and herbaceous vegetation has experienced a decline (−7.41%). Specifically, the expansion of hardwood forests has led to a potential increase in suitable habitat, resulting in a reduction of the effective distance between habitat patches. Patches in the west-central area exhibit higher betweenness centrality, indicating their importance as connectivity hubs. Connectivity metrics—including the Integral Index of Connectivity (IIC), Probability of Connectivity (PC), and Equivalent Connectivity (EC)—indicate an overall improvement in structural connectivity over the study period. Concurrently, fragmentation metrics such as the Splitting Index (SPLIT), effective mesh size (EM), edge length (EL), and edge density (ED) suggest a decrease in habitat fragmentation. Our analysis also demonstrates a correlation between citizen science observations from iNaturalist and the identified habitat patches and corridors, highlighting the value of such platforms in biodiversity monitoring despite inherent biases. The use of satellite remote sensing proved essential for obtaining detailed land cover information applicable to connectivity analyses, while graph-based connectivity metrics offer effective tools that require relatively low data input. These findings have important implications for land use and environmental planning, emphasizing the role of ecological connectivity and habitat preservation in informed environmental decision-making.

## Supplementary information

Appendix S1

## Data Availability

Data derived from this research, along with a description, can be found at the following link: https://zenodo.org/records/14731550
