# Supplementary material for: Past and Present in the Ecological Connectivity of Protected Areas Through Land Cover and Graph-Based Metrics
Source: Environ Manage. 2025 Jun 17;75(8):2116–35. doi: 10.1007/s00267-025-02206-1 (PMC12259746; doi:10.1007/s00267-025-02206-1)
Supplement: Supplementary file 1 — Appendix S1 [file 267_2025_2206_MOESM1_ESM.docx]

**Appendix S1**

| **Table 1** land cover classes reclassification | | |
| --- | --- | --- |
| SIOSE code | SIOSE class | Reclassified class |
| 111 | Historic town center | Artificial |
| 112 | Urban extensión |  |
| 113 | Discontinuous urban |  |
| 114 | Urban green zone |  |
| 121 | Agricultural and/or livestock facility |  |
| 123 | Mining extraction site |  |
| 130 | Industrial |  |
| 140 | Service or public facility |  |
| 150 | Agricultural settlement and orchard |  |
| 161 | Road or railway network |  |
| 162 | Port |  |
| 171 | Utility infrastructure |  |
| 172 | Waste infrastructure |  |
| 210 | Herbaceous crop |  |
| 220 | Greenhouse | Agricultural and herbaceous |
| 232 | Non-citrus orchard |  |
| 233 | Vineyard |  |
| 240 | Meadow |  |
| 250 | Crop combination |  |
| 260 | Crop combination with vegetation |  |
| 320 | Grassland or herbaceous vegetation |  |
| 330 | Shrubland |  |
| 340 | Vegetation combination |  |
| 352 | Rocky area |  |
| 354 | Bare soil |  |
| 413 | Salt marsh |  |
| 311 | Harwood forest | Hardwoods |
| 312 | Coniferous forest | Conifers |
| 313 | Mixed forest | - |
| 511 | Watercourse | Water bodies |
| 513 | Reservoir |  |
| 514 | Artificial water body |  |
| 515 | Sea |  |
| - | - | Eucalyptus |

| **Table 2** Confusion matrix for 2013. Predicted classes correspond to the horizontal axis while validated pixels are in the vertical axis. Diagonal represents correctly classified pixels. | | | | | | | |  |
| --- | --- | --- | --- | --- | --- | --- | --- | --- |
| 2013 | Agricultural and herbaceous | Artificial | Conifers | Hardwoods | Eucalyptus | Water bodies | Total |  |
| Agricultural and herbaceous | **976** | 6 | 2 | 1 | 11 | 0 | 996 |  |
| Artificial | 3 | **119** | 0 | 0 | 0 | 0 | 122 |  |
| Conifers | 23 | 0 | **91** | 0 | 16 | 0 | 130 |  |
| Hardwoods | 7 | 0 | 2 | **171** | 3 | 0 | 183 |  |
| Eucalyptus | 7 | 0 | 8 | 6 | **98** | 0 | 119 |  |
| Water bodies | 0 | 0 | 0 | 0 | 0 | **9** | 9 |  |
| Total | 1016 | 125 | 103 | 178 | 128 | 9 | 1559 |  |
| Commission error | 0.0201 | 0.0246 | 0.3000 | 0.0656 | 0.1765 | 0 |  |  |
|  |  |  |  |  |  |  |  |  |
| Omission error | 0.0394 | 0.0480 | 0.1165 | 0.0393 | 0.2344 | 0 |  |  |
| **Accuracy** |  |  |  |  |  |  | **0.939** |  |
| **Kappa** |  |  |  |  |  |  | **0.889** |  |

| **Table 3** Confusion matrix for 2023. Predicted classes correspond to the horizontal axis while validated pixels are in the vertical axis. Diagonal represents correctly classified pixels. | | | | | | | |  |
| --- | --- | --- | --- | --- | --- | --- | --- | --- |
| 2023 | Agricultural and herbaceous | Artificial | Conifers | Hardwoods | Eucalyptus | Water bodies | Total |  |
| Agricultural and herbaceous | **810** | 24 | 3 | 0 | 3 | 0 | 840 |  |
| Artificial | 2 | **76** | 0 | 0 | 0 | 0 | 78 |  |
| Conifers | 26 | 0 | **69** | 7 | 4 | 0 | 106 |  |
| Harwoods | 10 | 0 | 6 | **136** | 1 | 0 | 153 |  |
| Eucalyptus | 0 | 0 | 12 | 0 | **115** | 0 | 127 |  |
| Water bodies | 0 | 0 | 0 | 0 | 0 | **29** | 29 |  |
| Total | 848 | 100 | 90 | 143 | 123 | 29 | 1333 |  |
| Commission error | 0.0357 | 0.0256 | 0.3491 | 0.1111 | 0.0945 | 0 |  |  |
|  |  |  |  |  |  |  |  |  |
| Omission error | 0.0448 | 0.2400 | 0.2333 | 0.0490 | 0.0650 | 0 |  |  |
| **Accuracy** |  |  |  |  |  |  | **0.926** |  |
| **Kappa** |  |  |  |  |  |  | **0.871** |  |

**Fig. 1** iNaturalist records (N) temporary distribution.


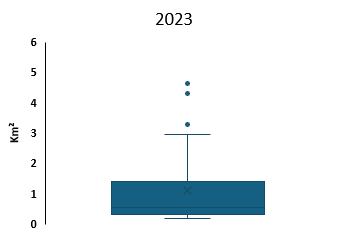

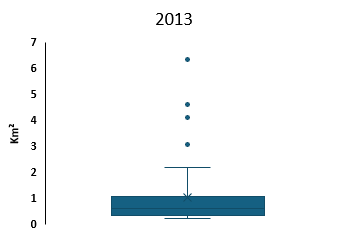


**Fig. 2** Boxplot with the surfaces (km^2^) of habitat patches.

**Fig. 3** Scatter plot with the BC and habitat patches surface (km^2^), blue values corresponding to 2013 while orange to 2023.


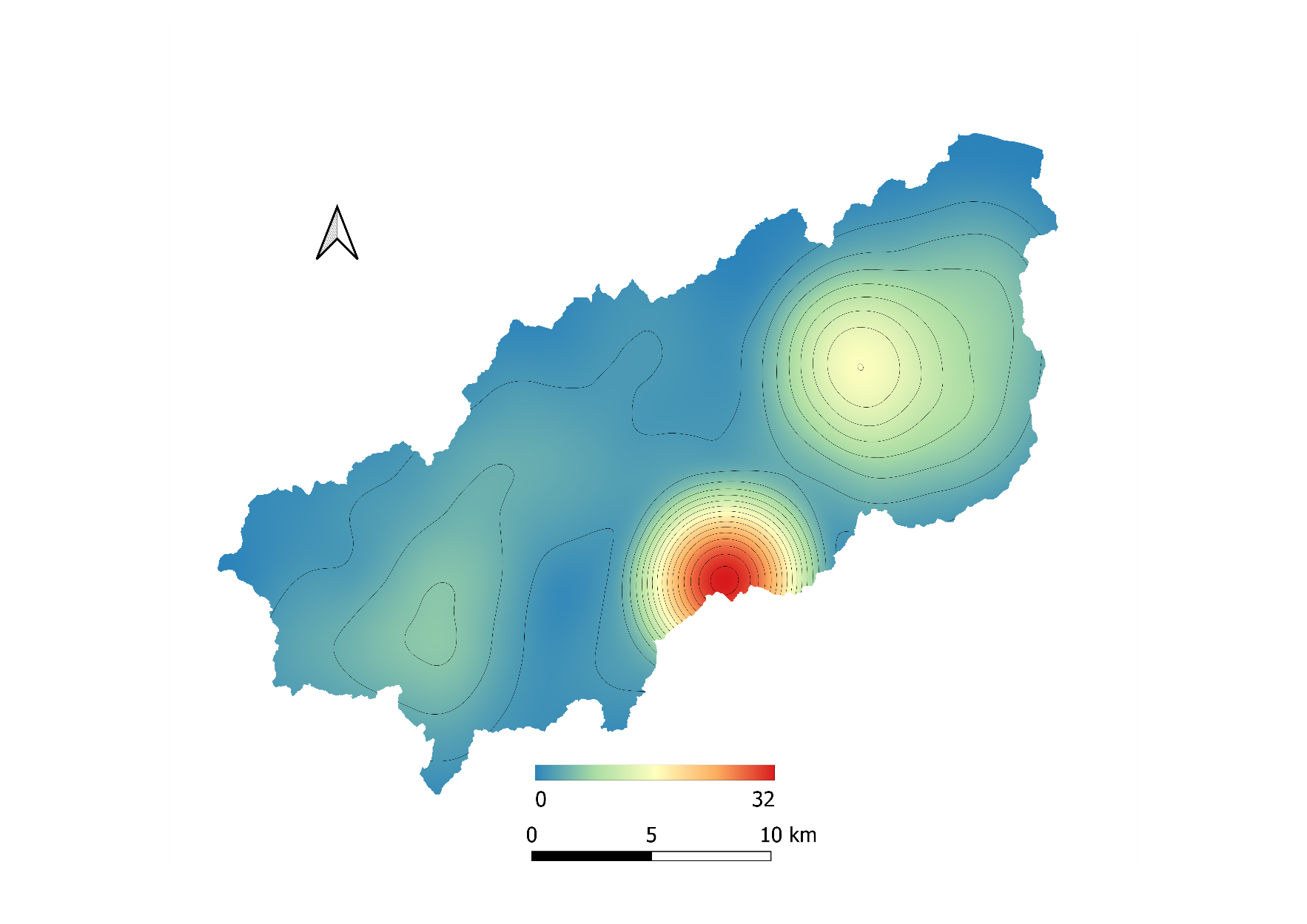


**Fig. 4** record distribution heatmap with an influence area of 5 km for each point, level lines represent a two record jump.


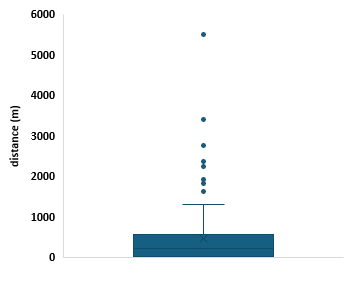


**Fig. 5** Minimum distance (m) between records location and habitat patches or paths identified by 2023.
